# A randomized controlled add-on trial of fluoxetine and cognitive behavioral therapy for help-seeking men with a sexual interest in children: presentation of the PARACHUTES trial protocol and initial feasibility data

**DOI:** 10.3389/fpsyt.2024.1448196

**Published:** 2024-08-09

**Authors:** Roberth Adebahr, Katarina Görts Öberg, Christoffer Rahm, Markus Byström, Charlotte Sparre, Adrian E. Desai Boström, Matteo Bottai, Jussi Jokinen, Josephine Savard

**Affiliations:** ^1^ Department of Clinical Sciences/Psychiatry, Umeå University, Umeå, Sweden; ^2^ ANOVA, Karolinska University Hospital, Stockholm, Sweden; ^3^ Department of Medicine, Karolinska Institutet, Stockholm, Sweden; ^4^ Centre for Psychiatry Research, Department of Clinical Neuroscience, Karolinska Institutet, & Stockholm Health Care Services, Region Stockholm, Stockholm, Sweden; ^5^ Division of Biostatistics, Institute of Environmental Medicine, Karolinska Institutet, Stockholm, Sweden

**Keywords:** child sexual abuse, paraphilic disorder, pedophilic disorder, preventive psychiatry, fluoxetine, therapy, cognitive behavioral

## Abstract

**Background:**

Sexual Interest in Children (SIC) is a major risk factor for sexual offending, yet clinical trials are sparse. The present protocol outlines a randomized controlled trial (RCT) that aims to investigate the effectiveness of fluoxetine and Cognitive Behavioral Therapy (CBT) in help-seeking men with SIC.

**Methods:**

Adult men contacting the Swedish telephone helpline PrevenTell are screened for inclusion and invited to further assessment on site. One hundred and eleven men with SIC (defined as DSM-5 pedophilic disorder or hebephilia) will be randomized (1:1:1 ratio) to receive one of three interventions for 14 weeks: (1) an internet-administered psychoeducational program (iPP), (2) iPP and the addition of fluoxetine 20-40 mg or (3) iPP and the addition of internet-administered CBT (iCBT). Exclusion criteria include severe psychiatric illness, contraindicating treatment and an elevated risk of committing hands-on sexual offences. Symptom intensity is assessed at baseline, pre-treatment, every other week for 12 weeks, and post treatment. The primary outcome measure is the Sexual Interest in Children: Current Assessment Scale (SIC: CAS) that quantifies sexual behaviors associated with SIC as well as perceived distress and impairment. Secondary outcomes include measures of dynamic risk-factors for committing sexual offences.

**Results:**

The data collected during the initial 20 months of recruitment were analyzed to predict the required number of individuals to be screened and estimate the probable length of the data collection phase. As of March 2022 to November 2023, 146 men have called PrevenTell and disclosed a sexual interest in minors. Following pre-screening, 110 men were excluded from participation in the trial. Current SSRI therapy was the primary reason for exclusion (n = 24; 22%), followed by an elevated risk of committing hands-on sexual offences (n = 14; 13%). Among the 31 men who underwent the screening procedure on site, 26 were allocated to either iPP, iPP+fluoxetine, or iPP+iCBT. The recruitment rate indicates that the trial will be concluded within the pre-estimated timeframe.

**Discussion:**

This is the first RCT of treatment with SSRI and iCBT in a population of help-seeking men with SIC. The significance of this trial and its methodological strengths and limitations are discussed.

## 1 Introduction

Pedophilic disorder (PeD), characterized by persistent sexual attraction to prepubescent children, is a risk factor for committing a first sexual offence as well as for recidivism (1–3). The estimated prevalence of a pedophilic sexual interest ranges between 0.3-3.8% in the adult male population, with an upper limit of 5%; the assumed prevalence in the adult female population is significantly lower (1). Sexual Interest in Children (SIC) includes pedophilic interest and interest in children who are entering puberty (ie, hebephilia) (4).

Given the considerable suffering and negative consequences associated with experiencing sexual abuse as a child, it is imperative to provide treatment to individuals at risk of committing such offences prior to their occurrence (5). The World Federation of Societies of Biological Psychiatry (WFSBP) provides guidelines for treatment of paraphilic disorders including PeD. The recommendations distinguish 5 levels of treatment depending on the risk for sexual offences, from low-risk individuals with mild paraphilic conditions to moderate and high-risk individuals and the most severe cases. The treatment algorithm includes Cognitive Behavioral Therapy (CBT) as the only intervention for mild cases (evidence level C/D), with the addition of Selective Serotonin Reuptake Inhibitors (SSRIs, eg, fluoxetine 40-60 mg/day or sertraline 200 mg/day, evidence level C) as the next step if satisfactory results are not obtained. Testosterone-lowering agents should be considered for individuals with moderate to high risk of committing sexual offences (6). Studies suggest that these agents reduce paraphilic sexual fantasies and outlet as well as the risk for committing Child Sexual Abuse (CSA) in help-seeking men with PeD. However, long-term treatment has documented adverse effects (eg, weight gain, gynecomastia, and bone density reduction), which necessitate a careful clinical evaluation of the benefits and potential risks before initiation, and thereby limit their use (7, 8).

Conversely, the adverse reactions associated with SSRIs (eg, headache, nausea, fatigue) are usually milder and typically decrease over time (9). Impaired sexual functioning is, however, a commonly reported adverse effect related to SSRI therapy and less likely to be transient; numerous studies suggest that more than 25% of individuals taking SSRIs experience sexual dysfunction as an adverse effect (10). When SSRIs are used to regulate problematic sexual behaviors, these agents significantly reduce desire for sexual behavior such as (1) frequency of masturbation and pornography use in non-offending men with compulsive sexual behaviors (11); (2) self-reported sexual behaviors in sexual offenders (12); and (3) self-reported frequency of unconventional sexual behaviors in men with paraphilic disorders (13).

The rationale for treating paraphilic disorders with SSRIs is based on the finding of reduced libido, as well as the beneficial effect on common psychiatric co-morbidities (eg, anxiety and depression). Moreover, it has been proposed that SSRIs may have a positive impact on the regulation of impulsive behavior (6).

Regarding studies specifically focused on examining the efficacy of SSRIs in the treatment of SIC, Winder, et al. (2014) conducted a trial in which men convicted of sexual offences (n = 49) (all but one convicted of child sexual abuse) were treated daily with fluoxetine 20-60 mg (n = 36), cyproterone acetate (CPA) 50-100 mg (n = 5), a combination of fluoxetine and CPA (n = 7) and a gonadotropin-releasing hormone agonist (n = 1). Results showed that all outcome measurements (sexual preoccupation, sexual compulsivity, and hypersexuality) were significantly reduced after 4 months of treatment in both the fluoxetine group and the anti-androgen group (14). In a retrospective study, Greenberg, et al. (1996) found a significant decrease in the severity of sexual fantasies in men (n = 58), of whom 71% were diagnosed with pedophilic disorder, after they received treatment with fluvoxamine, fluoxetine, or sertraline. During the 12-week study period, the fantasy severity decreased significantly between baseline and week 4, and between week 4 and week 8. There were no significant differences observed between the three SSRIs in terms of efficacy or reported adverse reactions (15). Additional support for the utilization of SSRIs in SIC is found in case studies (16, 17) however other case reports found that fluoxetine treatment did not lead to a significant change in pedophilic fantasies (18, 19).

None of the trials used a main outcome measure specifically assessing the change in behaviors associated with SIC and the study designs limit the conclusions that can be made. Research indicate that SSRI treatment could be a viable treatment option for the management of paraphilic disorders (20). To the extent of our knowledge, no randomized controlled design trial has evaluated SSRI treatment of SIC.

Although existing evidence indicates that treatment is generally effective in reducing sexual crime recidivism, the outcomes of psychological treatment programs specifically targeting child sexual offenders are less conclusive (21, 22). There is currently no firm evidence that psychological interventions reduce sexual re-offending in this subgroup (23). Regarding preventive psychological interventions, the German Preventive Project Dunkelfeld provides treatment to help-seeking at-risk individuals with SIC. In a non-randomized wait-list control evaluation of the psychological treatment program, treated individuals (n = 56) were compared to controls (n = 22) on pre-post self-report measures. The results suggested significant reduction in several dynamic risk factors for sexual violence (eg, offence-supportive attitudes and sexual dysregulation) (24). However, the robustness of the findings has been discussed and the statistical methods used in the original study have been criticized (25). In 2022, Lätth et al. published the results of a study which randomized individuals (n = 160) who used child sexual abuse material to receive either internet-delivered Cognitive Behavioral Therapy (iCBT) (n = 80) or a psychological placebo intervention (n = 80). Participants were anonymous and recruited from forums on Darknet. Results showed a significant decrease in viewing time, severity of content, and related behaviors in the active treatment group as well as support for the feasibility and safety of the treatment program (26).

In this trial, all participants receive psychoeducation – interventions provided to educate individuals about the nature of their disorder or illness. Psychoeducation has been proposed to reduce risk of relapse in patients with schizophrenia (27) and depression (28). However, the evidence for psychoeducation as a treatment intervention is limited by weak study designs and the fact that the concept itself is poorly defined. As Compulsive Sexual Behavior Disorder (CSBD), personality disorders, depression, and anxiety are common in men with SIC, the psychoeducational program used in this trial will not only address problematic sexual interests but also take common psychiatric comorbidity into account (29, 30).

Findings from preventive interventions show that a subpopulation of men at increased risk of committing sexual offences against children seek therapy (31, 32). This observation reveals an opportunity to proactively intervene before offences occur. The importance of developing evidence-based treatments for help-seeking men with SIC has been repeatedly emphasized. There is nevertheless a crucial lack of high quality trials (33). This protocol outlines the method and intervention framework for a randomized add-on-controlled trial evaluating treatment with fluoxetine and iCBT for help-seeking men with SIC, as well as preliminary results regarding feasibility.

### 1.1 Aim

#### 1.1.1 Primary objectives

The primary objective of the trial is to examine whether add-on therapy with fluoxetine or iCBT is more effective than a standalone psychoeducational program for the reduction of problematic sexual behaviors in help-seeking men with SIC.

#### 1.1.2 Secondary objectives

The secondary objectives include an investigation of whether add-on therapy is more effective than a psychoeducational program given alone for the reduction of (1) symptoms of psychiatric comorbidity, (2) perceived loneliness and improving quality of life, and (3) cognitive distortions and emotional congruence with children. For an account of all secondary objectives, see **Table 1**.

**Table 1:** Secondary objectives of the PARACHUTES trial.

Baseline
1. To investigate clinical and sociodemographic parameters associated with SIC.
2. To investigate biological markers associated with SIC.
Evaluation of treatment interventions
1. To evaluate whether add-on therapy with fluoxetine or iCBT is more effective than a standalone psychoeducational intervention to: (a) Reduce symptoms of psychiatric comorbidity. (b) Reduce perceived loneliness and improve quality of life. (c) Reduce cognitive distortions and emotional congruence with children.
2. To assess for any differences in drop-out rate and adherence between the treatment groups.
3. To assess adverse effects (fluoxetine) and negative effects (iCBT/iPP); to investigate any links between drop-out rates, reported adverse effects, and/or efficacy.
4. To compare any difference in treatment satisfaction between the treatment groups.
Evaluation of treatment (general)
1. To investigate whether there are any indications that clinical, psychosocial, or biological factors can predict treatment response, drop-out rate, and adherence to treatment.

## 2 Methods

### 2.1 Design

**Figure 1:**
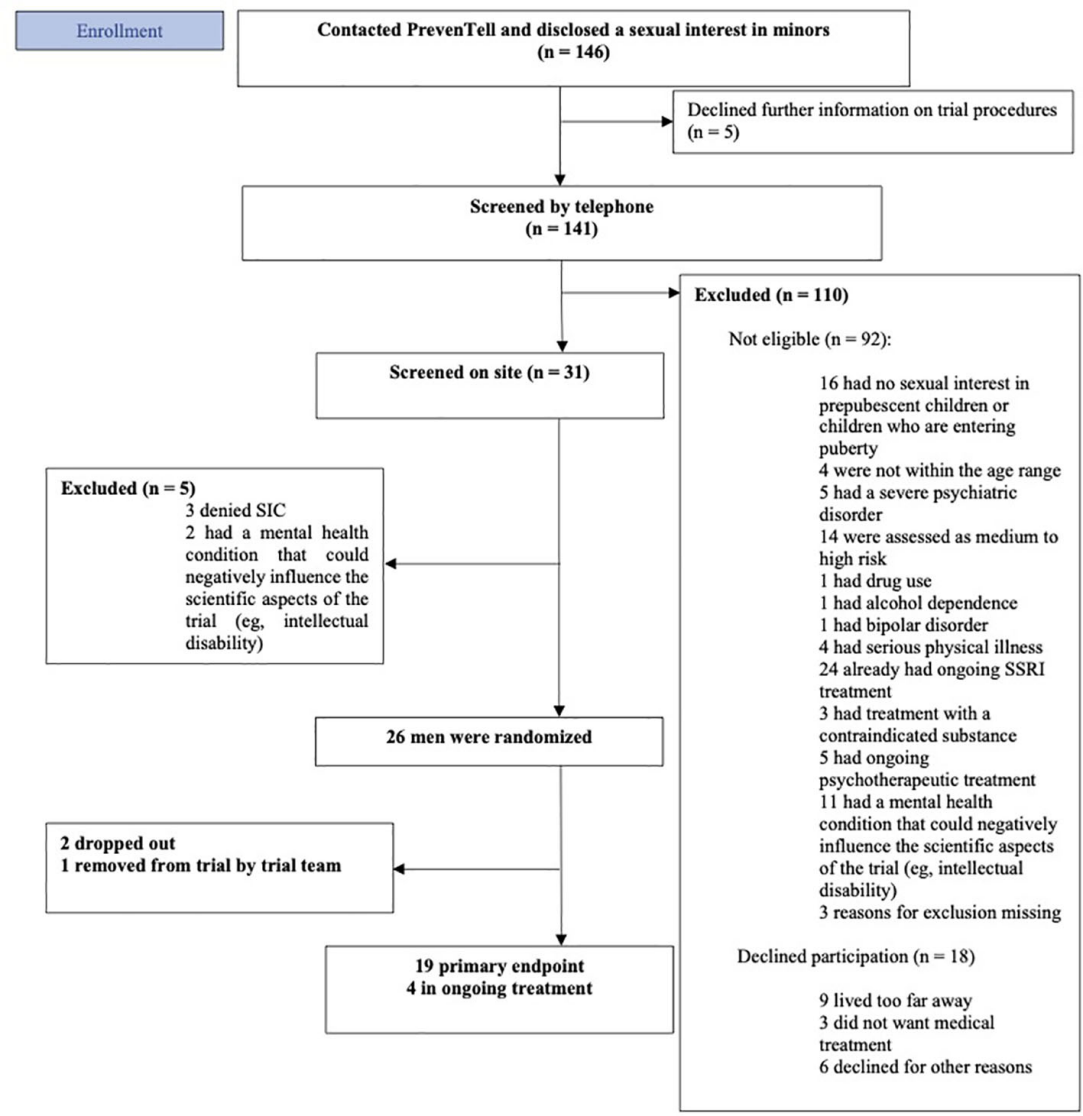
CONSORT diagram of the trial procedures.

The present clinical trial is an open randomized controlled add-on trial. Subjects will be randomized to one of the following conditions: (1) an internet-administered psychoeducational program, iPP (control intervention), (2) iPP and fluoxetine 20-40 mg daily (experimental intervention), or (3) iPP and iCBT (experimental intervention). The design controls for expectations of seeking treatment and participation in a trial, attention from the research team, and contact with a psychologist. The primary endpoint is at week 14. All participants will be encouraged to continue treatment after completing the trial procedures. Those randomized to the control intervention will be offered the iCBT program and pharmacological treatment if clinically warranted after the trial period **Figure 1** presents a CONSORT diagram illustrating each stage of the trial.

### 2.2 Site

The trial will be conducted at ANOVA, a center for sexual medicine, andrology, and trans medicine at Karolinska University Hospital, Stockholm, Sweden. Help-seeking men with SIC will be recruited consecutively through PrevenTell, a Swedish national helpline for unwanted or problematic sexuality operated by the ANOVA clinic (32).

### 2.3 Participants

The clinical trial will include men with SIC (n = 111), defined as DSM-5 pedophilic disorder or hebephilia, within the age range of 18 to 66 years. Individuals with severe psychiatric or physical illness or a moderate to high risk of committing hands-on sexual offences, as found after clinical evaluation by both psychiatrist and psychologist, will be excluded. For complete eligibility criteria, see **Table 2**.

**Table 2:** Eligibility criteria for the PARACHUTES trial.

Inclusion criteria
1. Signed informed consent form.
2. Aged 18-65 years.
3. Male.
4. Able to read and communicate in Swedish.
5. Have access to computer and internet.
6. Meet criteria for pedophilic disorder (per DSM-5) or hebephilia.
7. Agree to participate in all trial visits including providing blood and urine samples.
Exclusion criteria
1. Severe psychiatric disorder requiring immediate treatment such as current psychosis or severe depression.
2. Moderate to high risk for committing child sexual abuse: Contact-driven, loss of control over such impulses, and access to children.
3. Self-reported use of recreational drugs in the past month or positive drug verification analysis.
4. Alcohol dependence or risk consumption (> 14 units of alcohol per week) in the past month.
5. Participation in other trials or studies outside ANOVA.
6. Signs of hepatitis, elevated liver enzymes (> 3 times reference values), or a history of liver failure.
7. eGFR < 60 ml/min, signs or history of acute kidney failure.
8. Fasting plasma glucose ≥ 126 mg/dL (7.0 mmol/l).
9. Ongoing treatment in the past month with opioids or benzodiazepines. A restricted level of intermittent treatment is tolerated if it does not interfere with the trial treatment (as judged by trial psychiatrist).
10. Ongoing treatment in the past month with oral anticoagulants such as warfarin. Intermittent treatment (max. 15 doses per week) with NSAID (eg, ibuprofen) is tolerated.
11. Treatment with tamoxifen.
12. Bipolar disorder or history of hypomania.
13. Known heart disease such as angina pectoris, previous heart failure, or heart attack.
14. Other serious physical illness including diabetes mellitus, epilepsy, or known ocular hypertension.
15. Ongoing treatment with SSRI or previous hypersensitivity reaction.
16. Change of concurrent medication or dosage in the past three months regarding antidepressants, ADHD medication, mood stabilizers, antipsychotics, cortisone, testosterone, or dopamine precursors. Smaller adjustments may in some cases be acceptable (assessed by trial psychiatrist).
17. Ongoing pharmacological treatment with contraindicated substances (eg, MAOI, metoprolol).
18. Ongoing psychotherapeutic treatment.
19. Mental health condition that could negatively influence either the participant’s health or the scientific aspects of the trial (eg, intellectual disability).

DSM-5, The Diagnostic and Statistical Manual of Mental Disorders, Fifth Edition; NSAID, Non-steroid anti-inflammatory drug; MAOI, Monoamine oxidase inhibitor.

### 2.4 Procedures

ANOVA is the main healthcare provider in Sweden for individuals with self-identified problematic sexual behaviors (for example paraphilic disorders or compulsive sexual behavior disorder). There are multiple paths to become a patient at ANOVA: self-referral via PrevenTell, referral from another healthcare provider or from the prison and probation service. In order to assure the recruitment of help-seeking individuals, participants will largely be recruited via PrevenTell. However, individuals who have requested a referral to ANOVA through another healthcare provider may also be considered for participation. Individuals who reach out to PrevenTell due to concern about their own sexuality undergo a semi-structured interview. This interview focuses on identifying potential risk factors for engaging in sexual violence, including having a sexual interest in children. The interview has been described in detail elsewhere (32) PrevenTell personnel will request individuals who disclose any form of sexual interest in minors (< 18 years old) or behaviors associated with such interests, such as the use of Child Sexual Abuse Material (CSAM), to provide their consent for a follow-up call from the trial psychiatrist or psychologist. The purpose of this call is to provide additional information about the PARACHUTES trial (Pedophilia At Risk – Acute Treatment – E-therapy vs SSRI) and assess eligibility.

Eligible individuals will be invited to screening visits at the ANOVA clinic: two visits to a board-certified psychiatrist and two visits to a clinical psychologist, carried out over a maximum period of 21 days. On the first visit, the individual will receive written information about the trial and provide their informed consent. Thereafter, urine and blood samples for safety and specific research samples (blood samples for epigenetic and gene expression analysis) will be collected. Subsequently, a psychiatrist will obtain information on the individual’s sexual, medical, and psychiatric background and conduct a diagnostic interview based on DSM-5 criteria for PeD and hebephilia. The second visit to the psychiatrist involves a physical examination including blood pressure and heart and pulmonary auscultation, the Mini International Neuropsychiatric Interview 7.0.0, and Columbia Suicide Severity Rating Scale (34, 35).

The first visit to the psychologist includes filling out self-report measures on a secure web platform in private, with the psychologist available for assistance. During the second visit, the psychologist conducts a structured interview focusing on PeD or hebephilia (eg, age of onset, age and gender preference using Tanner scales, ongoing or previous illegal behaviors), compulsive sexuality, and co-occurring paraphilias (36, 37).

To control for observation bias, the psychiatrist and the psychologist then independently assess and confirm fulfillment of the diagnostic criteria for PeD or hebephilia as well as eligibility criteria.

**Figure 2:**
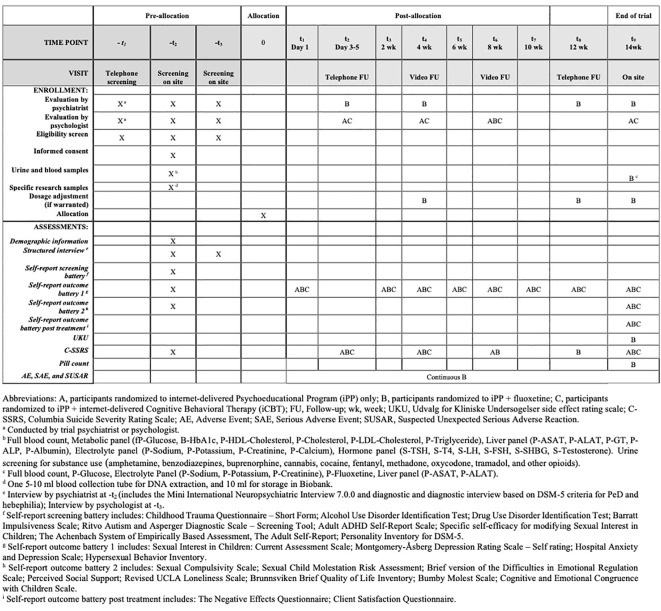
SPIRIT schedule of enrollment, interventions, and assessments.

For a detailed account of screening procedures, interventions, and assessments, see SPIRIT schedule, **Figure 2**.

### 2.5 Randomization

Participants will be randomized consecutively with a 1:1:1 allocation ratio. The research nurse will perform the randomization using opaque sealed envelopes provided by an external clinical trial unit, the Karolinska Trial Alliance. The allocation sequence is concealed to participants and the research team.

### 2.6 Interventions

#### 2.6.1 The internet-administered psychoeducational program

The program comprises 4 text modules which include information on paraphilic disorders and CSBD, depression, anxiety and crisis reactions, the consequences of being subjected to child sexual abuse, and the legal measures in Sweden aimed at safeguarding minors from such abuse (for a description of the contents, see **Table 3**). The program does not contain behavioral interventions or modification strategies. After each module, the participants will be asked to submit reflections on the topics highlighted in the module; these will be read by a designated psychologist who will then provide feedback. The participant will have access to the program for the whole duration of the trial, however the opportunity for direct feedback from a psychologist is restricted to a period of 4 weeks.

**Table 3:** Treatment content in the psychoeducational program and cognitive behavioral therapy program in the PARACHUTES trial.

	Intervention		
Treatment content	iPP	iPP + Fluoxetine	iPP + iCBT
Module 1			
Perspectives on how psychoeducation can support behavioral change	X	X	X
Information about treatment with fluoxetine		X	
Presentation of the overall treatment goals	X	X	X
Introduction to CBT			X
Worksheets:			
*Registration of problematic sexual behaviors*			X
*Identifying issues and setting goals*			X
*CBT triangle*			X
Reflection exercise:			
*Reflect on issues highlighted in the module*	X	X	
Module 2			
Description of SIC and other paraphilias/paraphilic disorders	X	X	X
Information about the consequences of victimization of sexual abuse	X	X	X
Information about why children can never give consent to sexual activity	X	X	X
Information about Swedish laws that criminalize the sexual abuse of minors	X	X	X
Worksheets:			
*Identify and challenge distorted beliefs about children’s sexuality*			X
*Registration of problematic sexual behaviors*			X
Reflection exercise			
*Reflect on issues highlighted in the module*	X	X	
Module 3			
Description of compulsive sexual behavior disorder	X	X	X
Information on the importance of consent for sexual activities between adults	X	X	X
Information about psychiatric comorbidity	X	X	X
Information about self-care interventions	X	X	X
Worksheets:			
*Registration of problematic sexual behaviors*			X
Reflection exercise:			
*Reflect on issues highlighted in the module*	X	X	
Module 4			
Identification of surpluses and deficits of sexual and non-sexual behaviors			X
Motivation			X
Perspectives on self-care, setbacks, and “when to ask for help”	X	X	
Worksheets:			
*Pros and cons of behavioral change*			X
*Inventory of behavioral surpluses and deficits*			X
*Registration of problematic sexual behaviors*			X
Reflection exercise:			
*Summary of interventions*	X	X	
Module 5			
Introduction to functional behavior analysis			X
Presentation of “chain analysis” as a tool for behavioral change			X
Worksheets:			
*Chain analysis*			X
*Registration of problematic sexual behaviors*			X
Module 6			
Psychoeducation about sexual urges			X
Psychoeducation about emotions			X
Psychoeducation about negative thought patterns			X
Worksheets:			
*Cognitive restructuring*			X
*Observing emotions*			X
*Registration of problematic sexual behaviors*			X
Module 7			
Mindfulness: techniques and practices			X
Information on how to create a personal risk card			X
Worksheets:			
*Risk card*			X
*Registration of problematic sexual behaviors*			X
Module 8			
Introduction to values and value-based goals			X
Introduction to behavioral experiments			X
Worksheets:			
*Identification of values “bullseye”*			X
*Goals based on values*			X
*Behavioral experiment*			X
*Registration of problematic sexual behaviors*			X
Module 9			
Social needs, social anxiety, and impairment in social functioning			X
Assertiveness and commonly used communication behavior styles			X
Social skills training			X
Worksheets:			
*Assessment of assertive communication skills*			X
*Behavioral experiment – “alternative communication style”*			X
*Registration of problematic sexual behaviors*			X
Module 10			
Perspectives on setbacks, relapse, and “when to ask for help”			X
Introduction to design a personal maintenance and relapse prevention program			X
Worksheets:			
*Maintenance and relapse prevention program*			X
*Summary of treatment*			X
*Registration of problematic sexual behaviors*			X

iPP, internet-delivered Psychoeducational Program; iCBT, internet-delivered Cognitive Behavioral Therapy; SIC, Sexual Interest in Children.

#### 2.6.2 Treatment with fluoxetine

Participants randomized to pharmacological treatment will start with 20 mg fluoxetine per day; the dose can be augmented to 40 mg per day after four weeks if tolerated and justified by clinical evaluation. The decision to choose fluoxetine was primarily driven by our team's extensive clinical expertise with this specific SSRI. Additionally, it was deemed to have pharmacological benefits, especially in the augmentation procedure. We considered that two possible dosages (20 mg and 40 mg) would make the study procedures easier, ensure comparable participant-healthcare provider interaction as in the other treatment groups, and enhance the interpretation of the findings. The trial drug will be supplied by the hospital pharmacy at Karolinska University Hospital. If the participant reports failure to take the medication for more than 5 consecutive days, he will be considered to have dropped out of the trial. Compliance is to be verified by blood sample testing (P-Fluoxetine) and pill count at end of trial (see **Figure 2**). In order to monitor and assess perceived adverse drug reactions, the UKU side effect rating scale (UKU) will be used (38). Serious adverse events and suspected unexpected serious adverse reactions will be managed and reported in accordance with the regulatory requirements of the Swedish Medical Products Agency. Participants have the option to continue treatment with fluoxetine after the end of the trial.

#### 2.6.3 The iCBT program

The iCBT program is an adapted version of an evaluated and published manual developed by the research team at ANOVA and draws on techniques from behavioral, cognitive, and third wave theoretical frameworks (39). The program comprises 10 modules administered in a fixed order and includes key features of CBT (for the contents, see **Table 3**). The contents of the treatment program specifically target the following risk factors for sexual recidivism: sexual preoccupation, sexualized coping, SIC and other paraphilias, emotional congruence with children, offence-supportive attitudes, self-regulation problems, and lack of relationships/feelings of loneliness (40, 41). Participants are expected to complete one module every week by returning homework assignments related to the topics covered in each module; a designated psychologist will give direct feedback on homework before the next module commences. All participants will have access to the program for the entire 14-week period, but access to direct support from the psychologist is limited to a period of 12 weeks.

#### 2.6.4 Follow-up

A secure web platform will be used for data collection and psychologist-participant communication. Participants will be instructed to log on to the web platform the day after randomization to access the first module of the treatment program. Every participant will be assigned an individual psychologist who will be responsible for providing feedback on reflection exercises (iPP and iPP+fluoxetine) and homework assignments (iCBT) between and during follow-up visits. Participants have the option to work at their own preferred speed during the treatment period, however they are encouraged to complete one module each week. The psychologist will be available to provide feedback via an email service on the web platform during two scheduled days each week. Participants assigned to the iPP+fluoxetine group will receive follow-up from a psychiatrist as outlined in the SPIRIT schedule shown in **Figure 2**. For all participants, the follow-up procedure consists of a phone call between day 3 and day 5, followed by a video meeting at 4 weeks, before the final visit on site at 14 weeks. Participants who are allocated to either iPP or iPP+fluoxetine receive an extra video follow-up at the 8-week mark.

### 2.7 Outcome measures

#### 2.7.1 Primary outcome measure: Sexual Interest in Children: Current Assessment Scale (SIC: CAS)

There is no gold standard questionnaire or assessment method for measuring change in problematic sexual behaviors in men with SIC. SIC: CAS is a self-report scale developed by the ANOVA research team and Umeå University. The scale is a modified version of the Hypersexual Disorder: Current Assessment Scale (HD: CAS) (42).

**Figure 3:**
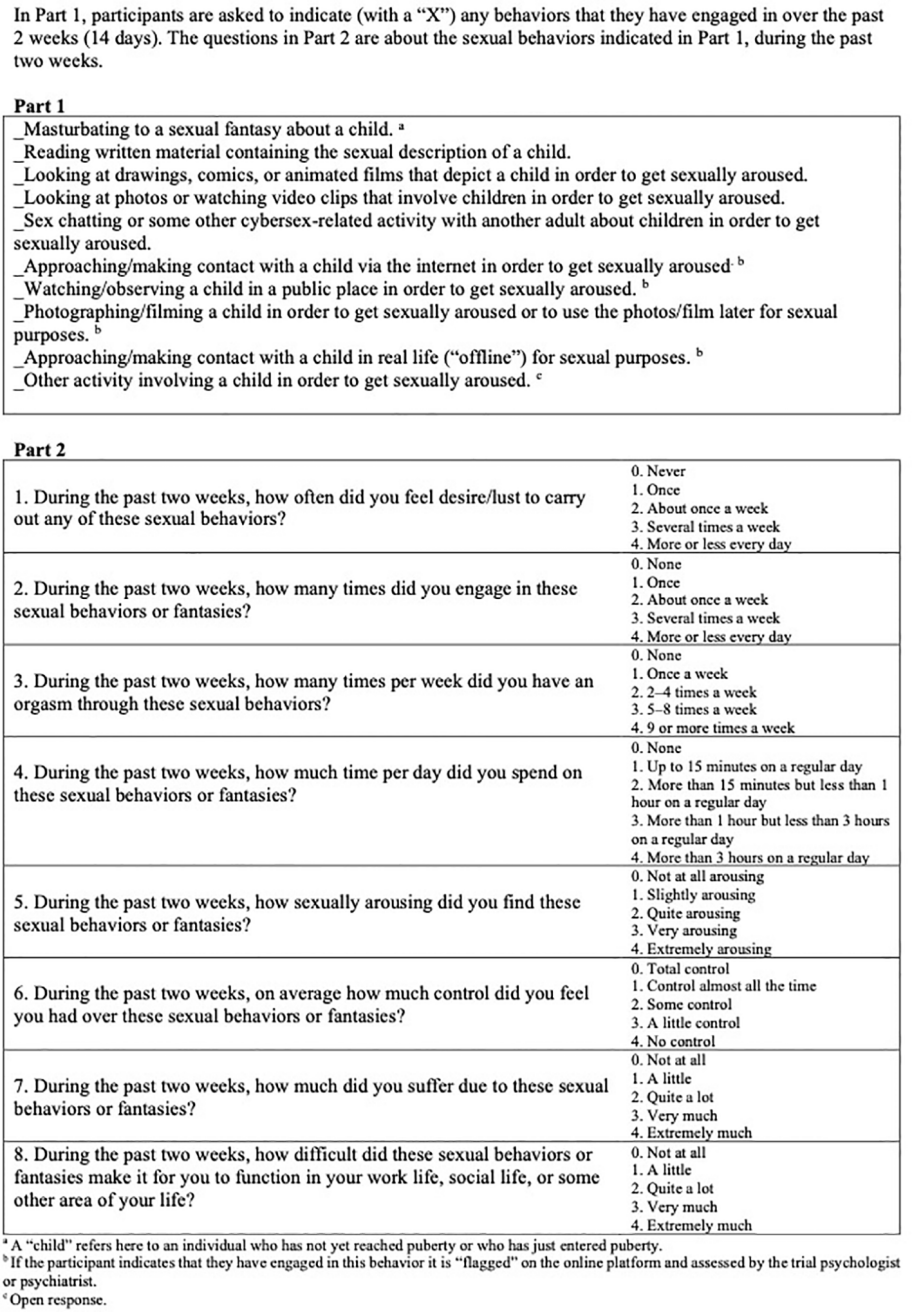
Sexual Interest in Children: Current Assessment Scale (SIC: CAS).

HD: CAS has been used in prior research conducted at ANOVA to evaluate both pharmacological and non-pharmacological interventions in men diagnosed with CSBD, with or without paraphilic interests (39, 43, 44). SIC: CAS was developed to measure changes in sexual behaviors as well as perceived distress, impairment, and loss of control associated with SIC during the previous 2 weeks. The scale comprises 2 parts. Part 1 covers behaviors related to SIC (eg, masturbation and watching animated material depicting children). In Part 2 the participant is asked to quantify and assess preoccupation with behaviors described in Part 1, as well as their impact on everyday functioning, and experienced distress in relation to these behaviors. Each item in Part 2 (1-8) is scored on a Likert scale (0-4 points) and the total score ranges from 0-32 points. All items can be found in **Figure 3**.

#### 2.7.2 Secondary outcome measures

**Figure 2** contains secondary outcome measures and time points. See **Supplementary Table 1** for a comprehensive description of the outcome measures.

### 2.8 Sample size calculation

We designed a randomized clinical trial comprising three groups: a control (iPP) and two active treatments (iPP+fluoxetine and iPP+iCBT). We compared each of the two active treatment groups with the control using two separate comparisons (iPP vs. iPP+fluoxetine, and iPP vs. iPP+iCBT). We used pilot data on HD: CAS from a previous CBT study in help-seeking men with hypersexual disorder to obtain estimates for the variance components required in the power calculations (39). We assumed that SIC: CAS would be psychometrically equivalent to HD: CAS. We estimated that it would suffice to enroll 110 participants to detect a difference of 0.29 points in SIC: CAS biweekly change in either one of two independent two-group comparisons with a random-intercept, random-slope mixed effects model with a significance level of 0.05 and a power of 80% (45, 46).

### 2.9 Statistical analysis

We will analyze the final data following an intention-to-treat principle. We will use mixed effect models as described in the previous section on the sample size calculations. If we observe major unbalances between the treatment groups in the distribution of the measured variables, these will be controlled for by introducing the corresponding variables in the regression models. We will evaluate possible differences in subpopulations of patients by introducing interactions terms with the relevant variables in the regression models. The mixed effects models will appropriately handle possible attrition of participants over the follow-up period under the assumption that the missing values caused by the loss to follow-up are missing at random. We will investigate the validity of this assumption by obtaining information on the causes of the attrition. The statistician performing the data analysis will be blind to the group allocation.

### 2.10 Ethical considerations

The trial has been approved by The Swedish Ethical Review Authority and The Swedish Medical Products Agency (ref no. 2021-02820). The clinical trial was registered before inclusion of the first trial participant and is accessible at http://www.clinicaltrialsregister.eu (Eudra-CT number: 2021-001249-11). The trial will be monitored by the Karolinska Trial Alliance.

While there is a pressing need to address the scarcity of high-quality studies on individuals with SIC, the choice of trial design and control group is not without ethical challenges. In the most serious situations, missing an opportunity to provide treatment may lead to the possible exposure of third parties to criminal offences. The treatment interventions included in this trial (fluoxetine and CBT) are recommended for individuals with paraphilic disorders at low risk of committing hands-on sexual offences (6). Individuals at a moderate to high risk of committing hands-on sexual offences, such as contact-driven men with access to children, will be excluded from participation and offered individualized treatment at the ANOVA clinic. It is also important to note that the participants included in this project are help-seeking individuals who express motivation to undergo treatment. The level of motivation may nonetheless vary throughout the trial period, thus necessitating careful monitoring of both problematic sexual behaviors and psychiatric symptoms. The ANOVA research group, along with clinical staff and clinic management, have extensive expertise in the assessment and treatment of patients at risk of committing sexual offences. In Sweden, healthcare professionals can breach patient confidentiality to report criminal offences serious enough to result in a minimum prison sentence of one year. Additionally, any violent and sexual offences committed against children can be reported, even if these offences do not meet the threshold of one year in prison. If there is any indication that a specific minor or group of minors is at risk of harm, healthcare professionals are obligated to report this to social services as specified in the Swedish Social Services Act. Participants will be informed about these exceptions to patient confidentially.

## 3 Results

Data from the first 20 months of recruitment were compiled to forecast the minimum required number of screened individuals, and the probable length of the data collection period. Between March 2022 and November 2023, 146 men have called the PrevenTell helpline and disclosed a sexual interest in children. Five men declined further information about trial procedures and were therefore not followed up. Trial staff pre-screened 141 men by telephone. Among these, 78% (n = 110) were excluded from the trial. Fifteen percent (n = 16) denied sexual interest in pre-pubescent children or children in the early stages of puberty. Current SSRI therapy was the primary reason for exclusion (n = 24; 22%), followed by an elevated risk of committing hands-on sexual offences (n = 14; 13%). Sixteen percent (n = 18) declined participation; the primary reason for declining to participate was the distance from their home to the clinic. See **Figure 1** for flowchart. Of the 31 men screened on site, 26 men were randomized to receive either iPP, iPP+fluoxetine, or iPP+iCBT.

## 4 Discussion

This will be the first randomized-controlled trial to evaluate treatment with SSRI and iCBT in men with SIC seeking treatment in a clinical context. The results will provide information on the effectiveness of these treatment options in reducing problematic sexual behaviors and risk factors for sexual violence in this population.

Approximately twenty percent of the men who undergo screening can be expected to be included in the trial. A common reason for declining participation in this trial is the geographical distance between the individual’s home and the clinic. The expenses associated with travel, accommodations, and income loss were considered too high in this group. Internet-administered psychological treatment could be a promising approach to reach these individuals. This is, nevertheless, only likely to be suitable for a particular subgroup of individuals; other interventions require the patient to be physically present. Furthermore, based on our clinical experience, on-site consultations are optimal, especially during the evaluation stage. This highlights the importance of also ensuring access to treatment in rural settings.

A considerable proportion of the men who underwent initial telephone screening were excluded from the trial due to current SSRI therapy; a possible interpretation is that SSRIs are ineffective or insufficient for some individuals and that these patients would potentially benefit from dose augmentation or additional interventions. The number of men with SIC who benefit from SSRI treatment and therefore do not seek further medical attention is currently unknown.

Moreover, 13% were excluded from trial participation because they were identified as having a risk of committing hands-on sexual offences. This aligns with past studies on help-seeking men with SIC, in which approximately 15% admitted to prior physical offences against minors (32).

It should be noted that the documented reasons for exclusion may not fully and precisely represent the excluded group. During the telephone screening, we initially assess whether the inclusion criteria are met. Subsequently, we assess the presence of serious psychiatric and physical illness, current medication, the risk of hands-on sexual offences, ongoing psychological therapy, and finally, any other mental health conditions that may disqualify participation, such as intellectual disability. Although there may be multiple grounds for excluding an individual, only the initial reason is documented.

This clinical trial has several limitations. Firstly, included participants are help-seeking men evaluated as low-risk for hands-on crime. The interventions are adapted accordingly, thus limiting the generalizability of the results to individuals with a low-risk profile. The efficacy of providing therapy in a forensic environment to reduce sexual offence recidivism in low-risk individuals has been debated (47). The participants in this trial are currently deemed to have a low risk of committing hands-on abuse. However, this does not imply that they do not pose significant risks of acting on their sexual interest in children in other ways. In the PrevenTell study, 63% (n = 215) of men with SIC reported CSAM use (32). Furthermore, research from the German Network *Kein Täter Warden* shows that help-seeking men have a substantial risk of sexual recidivism (48). We maintain, therefore, that provision of treatment is justifiable and necessary to prevent harm to children, in addition to the humanitarian aspect of alleviating personal suffering in men with SIC. Secondly, the open trial design and lack of placebo control is a significant limitation. A double-blinded placebo-controlled trial would have provided a higher level of evidence regarding the efficacy of treatment with fluoxetine. This was unfortunately not an alternative, partly due to lack of funding. Regarding psychotherapy, there is no consensus on how to best control for non-specific treatment effect (49).

Thirdly, our main outcome measure, SIC: CAS, is not validated; further investigation of the psychometric features would have been beneficial. It is, however, based on a measurement (HD: CAS) that has been shown in prior studies to detect treatment effects in men with CSBD with or without paraphilic disorders (39, 43, 44).

Lastly, the trial protocol duration of 14 weeks is a shortcoming because it only provides information on the short-term treatment effect. Regarding fluoxetine treatment, it is expected to reach steady state during the timeframe of four to eight weeks (9). While the optimal dosage of fluoxetine is unclear, the maximum dosage in this trial is 40 mg for feasibility reasons.

## 5 Conclusion

Our initial evaluation of the recruitment process shows that this randomized controlled trial protocol is feasible. Based on the current recruitment rate, the trial is projected to be concluded within the expected timeframe (the first quarter of 2028). If the results demonstrate that fluoxetine and iCBT treatment are tolerable and effective, this might serve as a motivation for men with SIC to proactively seek treatment. Additionally, clinicians will be provided with a pharmacological treatment option that has been extensively validated for other conditions, and psychotherapy provided over the internet that can be administered to patients who would otherwise encounter obstacles to healthcare access.
