# Supplementary material for: A randomized controlled add-on trial of fluoxetine and cognitive behavioral therapy for help-seeking men with a sexual interest in children: presentation of the PARACHUTES trial protocol and initial feasibility data
Source: Front Psychiatry. 2024 Aug 9;15:1448196. doi: 10.3389/fpsyt.2024.1448196 (PMC11341493; doi:10.3389/fpsyt.2024.1448196)
Supplement: Supplementary file 1 [file Table_1.docx]

| Supplementary Table 1. Description of trial measurement instruments in the PARACHUTES trial | | | |
| --- | --- | --- | --- |
| **Objective** | **Assessment** | **Measured parameter** | **Time point** |
| Assess ongoing and lifetime episodes of the most common psychiatric disorders including antisocial personality disorder (1). | Mini International Neuropsychiatric Interview v 7.0.0 (MINI) | Eligibility | Baseline |
| Screen for paraphilic disorders listed in DSM-5 and ICD-11 as well as hebephilia (2). | Långström Self-rating Sexual Interests (LASSIE) | Eligibility | Baseline |
| Identify at-risk alcohol consumption and indication of alcohol use disorder (3). | Alcohol Use Disorder Identification Test (AUDIT) | Eligibility | Baseline |
| Identify drug or substance use disorder (4). | Drug Use Disorder Identification Test (DUDIT) | Eligibility | Baseline |
| Identify different aspects of childhood abuse and neglect (5). | Childhood Trauma Questionnaire – Short Form (CTQ-SF) | Clinical characteristics | Baseline |
| Assess impulsivity (6). | Barratt Impulsiveness Scale (BIS-11) | Clinical characteristics | Baseline |
| Screen for symptoms indicative of autism spectrum disorder (7). | Ritvo Autism and Asperger Diagnostic Scale – Screening Tool (RAADS-14) | Clinical characteristics | Baseline |
| Screen for ADHD in adults (8). | The Adult ADHD Self-Report Scale – Screen (ASRS-v1.1 Part A) | Clinical characteristics | Baseline |
| Assess the individual’s own belief that it is possible to modify their sexual interest in children (9). | Specific self-efficacy for modifying Sexual Interest in Children (SSIC) | Clinical characteristics | Baseline |
| Screen for behavioral problems, emotional problems, social difficulties, and thought disorders (10). | The Achenbach System of Empirically Based Assessment, the Adult Self-Report (ASEBA-ASR) | Clinical characteristics | Baseline |
| Assess personality-related problems (11). | Personality Inventory for DSM-5 (PID-5) | Clinical characteristics | Baseline |
| Assess change in sexual behaviors and perceived distress, impairment, and loss of control associated with SIC during the previous 2 weeks. | Sexual Interest in Children: Current Assessment Scale (SIC: CAS) | Primary outcome | Baseline, pretreatment, every two weeks during the treatment period, and posttreatment |
| Assess change in depression severity (12). | Montgomery-Åsberg Depression Rating Scale – Self-rating (MADRS-S) | Outcome | Baseline, pretreatment, every two weeks during the treatment period, and posttreatment |
| Assess change of depression and anxiety symptoms (13). | Hospital Anxiety and Depression Scale (HADS) | Outcome | Baseline, pretreatment, every two weeks during the treatment period, and posttreatment |
| Assess change in compulsive sexual behaviors (14). | The Hypersexual Behavior Inventory-19 (HBI-19) | Outcome | Baseline, pretreatment, every two weeks during the treatment period, and posttreatment |
| Assess change in degree of sexual compulsivity (15). | Sexual Compulsivity Scale (SCS) | Outcome | Baseline and posttreatment |
| Assess change in (1) self-rated risk of child sexual abuse behaviors and (2) actual occurrence of child sexual abuse behaviors (16). | Sexual Child Molestation Risk Assessment (SChiMRA) | Outcome | Baseline and posttreatment |
| Assess change in emotion regulation difficulties (17). | Brief version of the Difficulties in Emotional Regulation Scale (DERS-16) | Outcome | Baseline and posttreatment |
| Assess change in the perception of social support from (1) family and (2) friends (18). | Perceived Social Support (PSS) | Outcome | Baseline and posttreatment |
| Assess change the subjective experience of loneliness (19). | Revised UCLA Loneliness Scale (R-UCLA) | Outcome | Baseline and posttreatment |
| Assess change in overall life satisfaction (20). | Brunnsviken Brief Quality of Life Inventory (BBQ) | Outcome | Baseline and posttreatment |
| Assess change in cognitive distortions in individuals who have committed sexual offences against children (21). | Bumby Molest Scale (BMS) | Outcome | Baseline and posttreatment |
| Assess change in cognitive and emotional congruence with children (22). | Cognitive and Emotional Congruence with Children Scale (C-ECWC) | Outcome | Baseline and posttreatment |
| Assess suicidal ideation and behavior throughout the trial (23). | Columbia Suicide Severity Rating Scale (C-SSRS) | Safety | Baseline, telephone follow-up, visit at 4 weeks, visit at 8 weeks, and posttreatment |
| Assess perceived adverse drug reactions (24). | UKU side effect rating scale (UKU) | Tolerability | Posttreatment |
| Assess negative effects of psychological treatment (25). | The Negative Effects Questionnaire (NEQ) | Tolerability | Posttreatment |
| Assess client satisfaction with psychosocial interventions (26). | Client Satisfaction Questionnaire (CSQ-8) | Outcome | Posttreatment |

1. Sheehan DV, Lecrubier Y, Sheehan KH, Amorim P, Janavs J, Weiller E, et al. The Mini-International Neuropsychiatric Interview (M.I.N.I.): the development and validation of a structured diagnostic psychiatric interview for DSM-IV and ICD-10. J Clin Psychiatry. 1998;59 Suppl 20:22-33;quiz 4-57.

2. Långström N. Långström Self-rating Sexual Interests (LASSIE) Unpublished questionnaire. Stockholm: Author 2010.

3. Saunders JB, Aasland OG, Babor TF, de la Fuente JR, Grant M. Development of the Alcohol Use Disorders Identification Test (AUDIT): WHO Collaborative Project on Early Detection of Persons with Harmful Alcohol Consumption--II. Addiction. 1993;88(6):791-804.

4. Berman AH, Bergman H, Palmstierna T, Schlyter F. Evaluation of the Drug Use Disorders Identification Test (DUDIT) in criminal justice and detoxification settings and in a Swedish population sample. Eur Addict Res. 2005;11(1):22-31.

5. Gerdner A, Allgulander C. Psychometric properties of the Swedish version of the Childhood Trauma Questionnaire-Short Form (CTQ-SF). Nord J Psychiatry. 2009;63(2):160-70.

6. Patton JH, Stanford MS, Barratt ES. Factor structure of the Barratt impulsiveness scale. J Clin Psychol. 1995;51(6):768-74.

7. Eriksson JM, Andersen LM, Bejerot S. RAADS-14 Screen: validity of a screening tool for autism spectrum disorder in an adult psychiatric population. Mol Autism. 2013;4(1):49.

8. Kessler RC, Adler L, Ames M, Demler O, Faraone S, Hiripi E, et al. The World Health Organization Adult ADHD Self-Report Scale (ASRS): a short screening scale for use in the general population. Psychol Med. 2005;35(2):245-56.

9. Fromberger P, Schröder S, Bauer L, Siegel B, Tozdan S, Briken P, et al. @myTabu - A Placebo Controlled Randomized Trial of a Guided Web-Based Intervention for Individuals Who Sexually Abused Children and Individuals Who Consumed Child Sexual Exploitation Material: A Clinical Study Protocol. Front Psychiatry. 2020;11:575464.

10. Achenbach TM, Ivanova MY, Rescorla LA. Empirically based assessment and taxonomy of psychopathology for ages 1½-90+ years: Developmental, multi-informant, and multicultural findings. Compr Psychiatry. 2017;79:4-18.

11. Krueger RF, Derringer J, Markon KE, Watson D, Skodol AE. Initial construction of a maladaptive personality trait model and inventory for DSM-5. Psychol Med. 2012;42(9):1879-90.

12. Svanborg P, Asberg M. A comparison between the Beck Depression Inventory (BDI) and the self-rating version of the Montgomery Asberg Depression Rating Scale (MADRS). J Affect Disord. 2001;64(2-3):203-16.

13. Lisspers J, Nygren A, Söderman E. Hospital Anxiety and Depression Scale (HAD): some psychometric data for a Swedish sample. Acta Psychiatr Scand. 1997;96(4):281-6.

14. Reid RC, Garos S, Carpenter BN. Reliability, Validity, and Psychometric Development of the Hypersexual Behavior Inventory in an Outpatient Sample of Men. Sexual Addiction & Compulsivity. 2011;18(1):30-51.

15. Kalichman SC, Johnson JR, Adair V, Rompa D, Multhauf K, Kelly JA. Sexual sensation seeking: scale development and predicting AIDS-risk behavior among homosexually active men. J Pers Assess. 1994;62(3):385-97.

16. Landgren V, Malki K, Bottai M, Arver S, Rahm C. Effect of Gonadotropin-Releasing Hormone Antagonist on Risk of Committing Child Sexual Abuse in Men With Pedophilic Disorder: A Randomized Clinical Trial. JAMA Psychiatry. 2020;77(9):897-905.

17. Bjureberg J, Ljotsson B, Tull MT, Hedman E, Sahlin H, Lundh LG, et al. Development and Validation of a Brief Version of the Difficulties in Emotion Regulation Scale: The DERS-16. J Psychopathol Behav Assess. 2016;38(2):284-96.

18. Ghaderi A, Scott B. Prevalence and psychological correlates of eating disorders among females aged 18-30 years in the general population. Acta Psychiatr Scand. 1999;99(4):261-6.

19. Russell D, Peplau LA, Cutrona CE. The revised UCLA Loneliness Scale: concurrent and discriminant validity evidence. J Pers Soc Psychol. 1980;39(3):472-80.

20. Lindner P, Frykheden O, Forsstrom D, Andersson E, Ljotsson B, Hedman E, et al. The Brunnsviken Brief Quality of Life Scale (BBQ): Development and Psychometric Evaluation. Cogn Behav Ther. 2016;45(3):182-95.

21. Arkowitz S, Vess J. An evaluation of the Bumby RAPE and MOLEST scales as measures of cognitive distortions with civilly committed sexual offenders. Sex Abuse. 2003;15(4):237-49.

22. Paquette S, McPhail IV. Construction and preliminary validation of the Cognitive and Emotional Congruence with Children (C-ECWC) Scale. Psychol Assess. 2020;32(8):739-51.

23. Posner K, Brown GK, Stanley B, Brent DA, Yershova KV, Oquendo MA, et al. The Columbia-Suicide Severity Rating Scale: initial validity and internal consistency findings from three multisite studies with adolescents and adults. Am J Psychiatry. 2011;168(12):1266-77.

24. Lindström E, Lewander T, Malm U, Malt UF, Lublin H, Ahlfors UG. Patient-rated versus clinician-rated side effects of drug treatment in schizophrenia. Clinical validation of a self-rating version of the UKU Side Effect Rating Scale (UKU-SERS-Pat). Nordic Journal of Psychiatry. 2011;55(sup44):5-69.

25. Rozental A, Kottorp A, Forsstrom D, Mansson K, Boettcher J, Andersson G, et al. The Negative Effects Questionnaire: psychometric properties of an instrument for assessing negative effects in psychological treatments. Behav Cogn Psychother. 2019;47(5):559-72.

26. Larsen DL, Attkisson CC, Hargreaves WA, Nguyen TD. Assessment of client/patient satisfaction: development of a general scale. Eval Program Plann. 1979;2(3):197-207.
